# Validity, Reliability, and Cultural Adaptability of the Arabic Cognitive Flexibility Scale (Ar-CFS) Among Saudi Arabians: A Two-Cohort Investigation

**DOI:** 10.3390/healthcare12212163

**Published:** 2024-10-30

**Authors:** Nasser M. AbuDujain, Abdullah AlDhuwaihy, Faisal Alshuwaier, Yazeed B. Alsulaim, Norah Aldahash, Saleh Aljarallah, Turky H. Almigbal, Abdullah A. Alrasheed, Mohammed A. Batais, Matthew M. Martin

**Affiliations:** 1 Department of Family and Community Medicine, College of Medicine, King Saud University, Riyadh P.O. Box 11495, Saudi Arabia; 2 College of Medicine, King Saud University, Riyadh P.O. Box 11495, Saudi Arabia; 3 Department of Communication Studies, West Virginia University, Morgantown, WV 26506, USA

**Keywords:** cognitive flexibility, validity, reliability, factor analysis, Arabic, Saudi Arabia

## Abstract

**Background/objectives:** Cognitive flexibility is the mental skill that allows a person to shift between different ideas or concepts and think about several concepts simultaneously. A commonly used tool to assess cognitive flexibility is the Cognitive Flexibility Scale (CFS). This study focused on translating and validating the CFS into Arabic, given the lack of existing Arabic tools for assessing cognitive flexibility. **Methods:** Conducted at King Saud University, Riyadh, between April and June 2024, the study employed random sampling and involved 529 participants, including 419 students and 110 patients. **Results:** The average participant age was 25.8 years, with females making up 56.9% of the sample. Participants completed the Arabic CFS, along with the Emotion Regulation Questionnaire (ERQ) and the Perceived Stress Scale (PSS), at two time points 3–6 weeks apart. Reliability was evaluated through internal consistency (Cronbach’s alpha = 0.8) and test–retest reliability (intraclass correlation coefficient = 0.82). Item analysis indicated that removing any single item did not significantly impact overall reliability, and inter-item correlations were strongest between items 4 and 6 (0.55) and items 6 and 7 (0.51). Validity was assessed through face, content, and construct validity, with factor analysis revealing a dominant single component accounting for 34.4% of the variance, confirming the scale’s unidimensionality. Content validity indices for all items exceeded 0.9 in terms of relevance, importance, simplicity, and clarity. The Arabic CFS demonstrated good construct validity, showing significant correlations with the ERQ’s reappraisal and suppression dimensions and the PSS. **Conclusion:** In conclusion, the Arabic version of the CFS is a valid and reliable tool for assessing cognitive flexibility in Arabic-speaking populations. This tool will be valuable in both clinical and research settings within Saudi Arabia, offering a robust instrument for evaluating cognitive flexibility.

## 1. Introduction:

In the field of psychological assessment, it is crucial to ensure that the tools used to measure cognitive and behavioral attributes are culturally and linguistically appropriate for the populations under study [1,2]. Patient-reported outcomes (PROs) have become a cornerstone in evaluating cognitive and behavioral health because of their ability to capture the patient’s self-assessment of their mental and emotional state without external bias [3]. PROs are quantified using patient-reported outcome measures (PROMs) [4]. One of the most widely used PROM scales is the Cognitive Flexibility Scale (CFS) [5]. It assesses the ability of an individual to adapt cognitive processes in response to changing environmental demands. However, the absence of a validated and culturally adapted version of the CFS for Arabic-speaking populations poses a significant gap in psychological assessment tools available in the region.

Cognitive flexibility is the mental ability to switch between thinking about two different concepts and thinking about multiple concepts simultaneously [6,7,8]. It represents a person’s ability to adapt cognitive processing strategies to face new and unexpected environmental conditions. It is a critical executive function that has a significant impact on various psychological processes and overall mental health. Therefore, accurate measurement of cognitive flexibility across diverse cultural contexts is imperative. PROMs are used to evaluate cognitive flexibility in patients [9]. They provide clinicians with valuable insights into a patient’s cognitive functioning, emotional well-being, and overall quality of life. They are crucial in tracking cognitive changes over time, identifying deficits, and determining the most appropriate and effective therapeutic intervention to undertake [9,10].

Developed by Martin and Rubin in 1995, the CFS has 12 items, each rated on a 6-point Likert scale where responses range from 1 (“strongly disagree”) to 6 (“strongly agree”) [11]. The tool captures the individual’s ability to adapt to changing situations and their flexibility in problem-solving contexts. The CFS’s validity across various populations and cultures is solid, having shown high internal consistency and construct validity. Martin and colleagues, using samples from the United States, have found that individuals higher in cognitive flexibility are more assertive and responsive [12], willing to collaborate [13], conversationally sensitive [14], and self-compassionate [15]. Previous research has demonstrated the importance of culturally adapting psychological scales to enhance their reliability and validity. Different languages, including Turkish, Japanese, and Spanish, have translated and validated cognitive flexibility tests, including the CFS [16,17,18]. Numerous studies have applied the CFS to investigate cognitive flexibility in relation to various psychological conditions such as anxiety, Down syndrome, and depression [19,20,21,22,23,24,25].

To the best of our knowledge, there is no previous evidence of a validated CFS in Arabic. Thus, the development of an Arabic version of the CFS (Ar-CFS) is crucial for its application in clinical and research settings within Arabic-speaking populations. Translation will ensure that the scale is linguistically accurate and culturally relevant, reflecting the specific cognitive and behavioral nuances of the Arabic-speaking population.

## 2. Methodology

### 2.1. Study Design, Participants, Setting

A quantitative, analytical, cross-sectional study was conducted at King Saud University in Riyadh, Saudi Arabia, between April 2024 and June 2024. It recruited participants from two locations within the university: (1) the College of Medicine [to involve medical students] and (2) the Family Medicine clinics at the King Saud University Medical City [to involve patients following up for different conditions]. The study targeted fluent adult Arabic speakers. The only exclusion criterion was non-Arabic speakers.

### 2.2. Questionnaire

Participants completed an electronic survey consisting of four sections: (1) demographic information, (2) type of participant, (3) the Cognitive Flexibility Scale, and (4) the Emotion Regulation Questionnaire and the Perceived Stress Scale. The demographic section gathered data on age, gender, and monthly income. One question asked the participants if they were a patient following up in the family medicine clinics or a medical student studying in the College of Medicine. The final section included the Arabic-translated version of the CFS, followed by the Arabic-validated ERQ and PSS.

#### 2.2.1. Cognitive Flexibility Scale

The CFS was developed by Martin and Rubin in 1995 [11,12]. The CFS is a 12-item, 6-point Likert scale that assesses an individual’s cognitive flexibility. The 6-point response scale for the CFS ranges from “strongly disagree” to “strongly agree”. Four of the 12 items are reverse-scored, including items 2, 3, 5, and 10. This scale measures a person’s awareness that options and alternatives are available in any given situation, willingness to be flexible and adapt to the situation, and self-efficacy in being flexible. It shows good psychometric properties with a coefficient alpha of 0.81 [11,12].

#### 2.2.2. Emotion Regulation Questionnaire

Dr. Gross and colleagues developed the ERQ in 2003 to evaluate individual differences in two domains: (1) cognitive reappraisal and (2) expressive suppression [26]. Cognitive reappraisal is the process in which individuals reconsider their perspective on a particular situation in order to adapt their emotional response, whereas expressive suppression is the process in which individuals consciously inhibit their emotional expression after the emotional response has already been initiated. It comprises 10 items, isolated into two subscales: cognitive reappraisal, which has 6 questions, and expressive suppression, which has 4 questions. Each question is scored on a 7-point Likert scale, ranging from 1 (strongly disagree) to 7 (strongly agree). In our study, we used an Arabic-translated version of the ERQ developed and validated in 2021 [27]. The Arabic translation showed good validity by averaging 0.79 for reappraisal and 0.73 for suppression. Test–retest reliability across 3 months was 0.69 for both scales [27].

#### 2.2.3. Perceived Stress Scale

The PSS was developed by Cohen et al. in the early 1980s to assess the perception of stress in individuals’ lives [28]. Through a meticulous process of item generation, reduction, validation, and refinement, the final scale comprises 10 items that evaluate the degree to which situations in one’s life are appraised as stressful. Each item is scored on a 5-point Likert scale ranging from 0 (never) to 4 (very often), with total scores ranging from 0 to 40. Its psychometric properties, including high reliability and validity, have been extensively studied and validated. Measures of validity include construct validity, which confirms that the PSS accurately measures the concept of perceived stress, and criterion validity, which demonstrates that the PSS correlates well with other established measures of stress. Reliability measures include internal consistency, typically indicated by a Cronbach’s alpha of 0.78, showing that the PSS produces stable results over time [28].

Almadi et al. translated the PSS into Arabic in 2012, ensuring that the scale was culturally appropriate and retained its reliability and validity in Arabic-speaking populations. Evaluation revealed that Cronbach’s alpha coefficients of the translated PSS were 0.8, and the test–retest reliability had an intraclass correlation coefficient of 0.90 [29].

### 2.3. Instrument Translation Process

We followed a thorough translation method, as recommended by Sousa et al. [30]. First, we obtained permission from the tool’s original developer to translate the scale. The tool was then given to two independent, certified professional translators for forward translation from English to Arabic. The first translation, conducted by a physician with a medical background, was labelled TL-1, and the second, conducted by a physician with a medical background, was labeled TL-2.

After completing the forward translations, authors N.A. and S.A. reviewed and compared both versions in detail to ensure consistency in meaning. They then merged the translations into a single document labelled PI-TL. This PI-TL version was given to a third independent translator, who was not involved in the forward translation, for backward translation from Arabic to English. This version was labeled B-TL 1. The original developer, M.M., reviewed the B-TL 1 translation to ensure it accurately reflected the original content. This resulted in a consensus and the creation of the pre-final Arabic version of the CFS, labelled P-FTL.

After creating the pre-final Ar-CFS, we assessed its face validity by presenting it to 23 fluent Arabic speakers. They were asked to review each item for simplicity and clarity. A few minor, syntax-related suggestions were provided and incorporated into the pre-final version.

### 2.4. Psychometric Analysis for Validity and Reliability

Before finalizing the Ar-CFS, we conducted a content validity assessment with a panel of 11 subject matter experts (SMEs), including eight family physicians, two psychiatrists, and one psychologist. Each SME evaluated every item across four domains—simplicity, relevance, importance, and clarity—using a four-point Likert scale (strongly agree, somewhat agree, maybe, strongly disagree). The panel’s feedback was incorporated into the scale, resulting in the final version of the Ar-CFS, labelled FTL.

To evaluate construct validity, we performed an exploratory factor analysis (EFA) to assess convergent and structural validity as well as correlation with the Arabic versions of the ERQ and PSS. For reliability testing, we used the test–retest method, asking all participants to complete the Ar-CFS again three to six weeks after their initial responses to measure stability.

### 2.5. Ethical Considerations

In February 2024, the Institutional Review Board at the College of Medicine, King Saud University, granted ethical approval (No. E-24-8610, Ref. No. 24/1140/IRB). Participants provided electronic consent after being informed about the study’s purpose, expected duration, and principal investigator’s contact details.

### 2.6. Statistical Analysis

Statistical analyses were conducted using RStudio software (R version 4.3.1). Descriptive statistics were calculated to summarize the demographic characteristics of the study population, including mean and standard deviation for continuous variables and frequencies (percentages) for categorical variables. An exploratory factor analysis (EFA) was performed using the promax rotation method to identify the underlying factor structure of the Cognitive Flexibility Scale (CFS). A confirmatory factor analysis (CFA) was conducted to validate the factor structure identified in the EFA. To evaluate global fit of the measurement model, we inspected the scaled model chi square, standardized root mean square residual (SRMR), comparative fit index (CFI), and root mean square error of approximation (RMSEA) with its 90% confidence interval. Local fit was examined by inspecting correlation residuals in the correlational metric. The internal consistency of the CFS was evaluated using Cronbach’s alpha and test–retest reliability with the intraclass correlation coefficient (ICC).

Content validity was assessed by calculating the individual relevance index/content validity index (I-CVI), individual simplicity index (ISI), individual importance index (III), and individual clarity index (ICI). Construct validity was assessed by examining the Pearson correlation coefficients between the CFS scores and related constructs, including the Emotion Regulation Questionnaire and the Perceived Stress Scale. Bivariate correlations between cognitive flexibility and emotion regulation, and cognitive flexibility and perceived stress were analyzed and visualized using scatter plots. Furthermore, an inter-item correlation matrix was used to evaluate the correlations between each pair of items within the scale, allowing us to assess the relationships among the items designed to measure cognitive flexibility. The analysis involved calculating the correlation coefficients, which provided insights into the degree of association between items. High correlations among certain items indicated that they effectively measured similar aspects of cognitive flexibility, supporting the scale’s construct validity. All statistical tests were two-tailed, and a *p*-value of less than 0.05 was considered statistically significant.

## 3. Results

The study included 529 participants, comprising 419 students and 110 patients. The mean age was 25.8 years overall, with 21.5 years among students and 42.5 years among patients. The gender distribution was 43.1% male and 56.9% female overall. The monthly income distribution showed that 57.3% had an income of less than SAR 2000 (USD 533). More details about the demographic characteristics are provided in Table 1.

Table 2 analyzes the internal consistency of the Arabic Cognitive Flexibility Scale by examining the impact of each item on the overall scale if removed. Items generally exhibited moderate corrected item–total correlations, suggesting that each contributed effectively to the overall construct. Items 6, 7, and 12 stood out with particularly strong correlations, reinforcing their importance in measuring cognitive flexibility. The variance observed when each item was deleted indicated that the scale maintained its integrity across items, ensuring reliable measurement. The Arabic Cognitive Flexibility Scale had a Cronbach’s alpha value of 0.80, meaning that all 12 items were fairly consistent. Each item’s influence on the overall reliability was assessed, with item deletion showing minor fluctuations in Alpha, suggesting that all items contributed positively to the scale’s reliability; however, Cronbach’s alpha values remained above 0.70 for all items. The test–retest reliability of 36 individuals, reflected by an intraclass correlation of 0.82, with an internal consistency of 0.85, indicated that the scale had strong stability over time, confirming its reliability as a tool for measuring cognitive flexibility in Arabic-speaking populations.

Table 3 displays the inter-item correlation matrix for the Ar-CFS questionnaire, showing the correlations between each pair of items. The correlations ranged from 0.08 to 0.55, indicating varying degrees of association between the items. Notable correlations included a strong correlation between item 4 (“I can find workable solutions to seemingly unsolvable problems”) and item 6 (“I am willing to work at creative solutions to problems”) with a value of 0.55, and between item 6 and item 7 (“In any given situation I am able to act appropriately”) with a value of 0.51. Other significant correlations included item 7 and item 8 (“My behavior is a result of conscious decisions that I make”) with a value of 0.50, and item 1 (“I can communicate an idea in many different ways”) showing moderate correlations with item 6 (0.41), item 7 (0.42), and item 9 (“I have many possible ways of behaving in any given situation”) at 0.39. The lowest correlation observed was between item 2 (“I avoid new and unusual situations”) and item 11 (“I am willing to listen and consider alternatives for handling a problem”) at 0.08, suggesting a weak relationship between these items.

**Figure 1:**
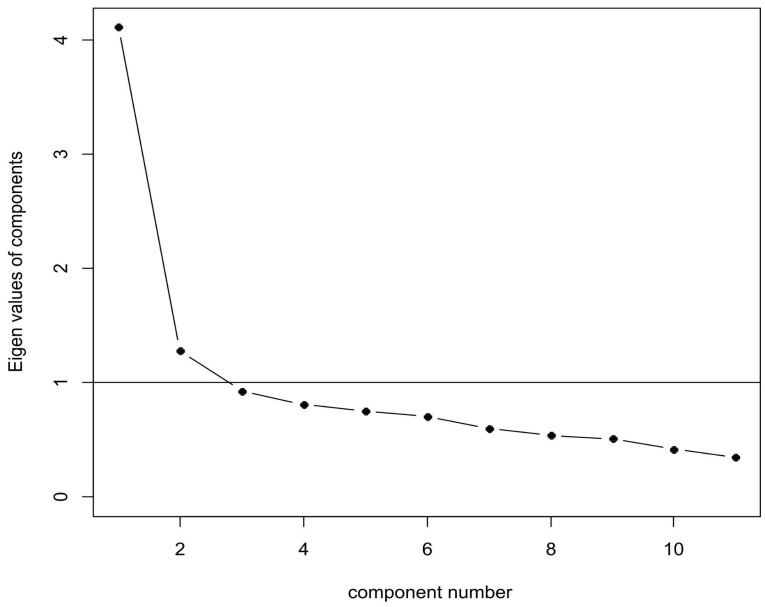
A scree plot of eigenvalues from the factor analysis.

The principal component analysis (PCA) delineated the underlying structure of the Arabic Cognitive Flexibility Scale, revealing a dominant first component that accounted for 34.373% of the total variance. This heavy load on a single component suggests that cognitive flexibility, as operationalized by this scale, was primarily a unidimensional construct. The additional 12% variance captured by the second component hinted at a possible secondary dimension, perhaps related to contextual or situational flexibility. However, this remained subordinate to the dominant trait captured by the first component (Figure 1). Using the medical student sample, a CFA for the Cognitive Flexibility Scale demonstrated marginal global fit: x^2^ (54) = 186.722, *p* < 0.001, SRMR = 0.059, CFI = 0.852, RMSEA = 0.077 (90% CI: [0.065, 0.089]). Local fit indices demonstrated correlation residuals ranging from [−0.090] to [0.169]. Given these global and local fit values, the measurement model needed respecification. To probe potential model misspecification, modification indices were consulted. Modification indices indicated that item 4 and item 6 required a residual correlation to improve model fit. A closer inspection of these items revealed that both items had similar language related to finding solutions to a problem. A respecified CFA for the Cognitive Flexibility Scale including the correlation error of items 4 and 6 demonstrated marginal global fit: x^2^ (53) = 154.770, *p* < 0.001, SRMR = 0.055, CFI = 0.886, RMSEA = 0.068 (90% CI: [0.055, 0.080]). Local fit indices demonstrated correlation residuals ranging from [−0.097] to [0.167].

After this respecification, modification indices flagged an additional residual correlation between items 3 and 10 that would further improve model fit. Both items were negatively worded about one’s ability in decision-making. A respecified CFA for the Cognitive Flexibility Scale including the correlation error of items 4 and 6, as well as the correlation error of items 3 and 10, demonstrated acceptable global fit: x^2^ (52) = 139.020, *p* < 0.001, SRMR = 0.052, CFI = 0.903, RMSEA = 0.063 (90% CI: [0.051, 0.076]). Local fit indices demonstrated correlation residuals ranging from [−0.111] to [0.176]. The PCA results emphasized the scale’s efficacy in distilling the cognitive flexibility construct into a concise, primarily unidimensional measure. The rotated component matrix further elucidated the scale’s factor structure (Table 4), with the majority of items demonstrating high loadings on the primary component. This suggests that the items were well-aligned with the overarching construct of cognitive flexibility, particularly those items emphasizing adaptive problem-solving and behavioral versatility, such as “I am willing to work at creative solutions to problems” (loading = 0.751) and “I have the self-confidence necessary to try different ways of behaving” (loading = 0.728). The consistency of these loadings across items underscores the scale’s capacity to coherently measure cognitive flexibility as a singular construct despite the inherent complexity of the concept.

In Table 5, the construct validity of the Ar-CFS is substantiated by its correlations with related psychological constructs, notably the ERQ and the PSS. The correlation with the reappraisal dimension of the ERQ was 0.12 (95% CI: 0.03 to 0.20, *p* = 0.007) for the overall participants, 0.12 (95% CI: 0.01 to 0.21, *p* = 0.021) among students, and 0.14 (95% CI: −0.05 to 0.32, *p* = 0.164 among patients, indicating statistically significant positive correlations among the overall participants and students. The correlation with the suppression dimension of the ERQ was significantly negative among the overall cohort with r = −0.11 (95% CI: −0.02 to −0.03, *p* = 0.011); however, the correlation was not significant among students (r = −0.11, 95% CI: -0.19 to 0.01, *p* = 0.056) or patients (r = −0.12, 95% CI: −0.33 to 0.04, *p* = 0.220). The correlation with the PSS was −0.45 (95% CI: −0.52 to −0.38, *p* < 0.001) for the overall participants, −0.48 (95% CI: −0.56 to −0.41, *p* < 0.001) among students, and −0.25 (95% CI: −0.43 to −0.07, *p* = 0.010) among patients, indicating significant negative correlations.

## 4. Discussion

The primary objective of our study was to evaluate the reliability, validity, and cross-cultural adaptability of the Ar-CFS. This involved internal consistency stability, inter-item correlation matrix analysis, and comparing the Ar-CFS with other established tools, such as the PSS and the ERQ. In the process, we verified its usefulness in medical and academic samples while affirming that it is applicable across various populations, including the Arab-speaking demographic.

Our sample’s internal consistency score was 0.80, which is a good level. It also matched up well with the original study by Martin et al. (0.81) [11], as well as with the Japanese (0.84), Turkish (0.74), and Spanish (0.81) studies [16,17,18]. Additionally, we evaluated the interclass correlation coefficient by administering the Ar-CFS to students on two separate occasions, spaced 3 to 6 weeks apart, unveiling an ICC of 0.82, suggesting that the tool produces stable and consistent results over time, further supporting its reliability. This aligns with the original study by Martin and Rubin [11], which revealed a reproducibility of 0.83. The same applies to the Japanese study (0.69) [17], which revealed an acceptable test–retest, and the Turkish study (0.98) [16], which produced an excellent test–retest.

Face, content, construct validity measures, and exploratory factor analysis were used to assess structural validity. The content validity indices, which assessed importance, simplicity, relevance, and clarity, showed an excellent score of 0.9 and above across all indices. An excellent S-CVI is accounted for when it exceeds 0.8 or 0.9 [31]. This is particularly significant as content validity was not assessed in other validated languages [16,17,18].

Exploratory factor analysis revealed a dominant unidimensional structure in which a primary component accounted for 34.37% of the total variance, with a hint of a secondary contextual or situational flexibility dimension. Therefore, in line with existing research findings, this study established that cognitive flexibility is primarily a singular construct measured by the Ar-CFS. This analysis aligns with the Japanese validation conducted by Oshiro et al. [17], which found a dominant one-factor Kaiser’s criterion with eigenvalues over 1.0 suggesting the possibility of a two-factor solution, which accounted for 35.5% of the variance for the 12-item measure. The Ar-CFS’s strong loadings on this primary component underscore its effectiveness in capturing the core aspects of cognitive flexibility.

Significant correlations with related psychological constructs confirmed the construct’s validity. There was a significant correlation between the Ar-CFS and the cognitive reappraisal domain for the ERQ (0.12, *p* = 0.007). Cognitive reappraisal and the CFS both measure the same construct, which is an approach that focuses on modifying how a potential trigger is interpreted, either intensifying or reducing its emotional impact. A positive correlation, in turn, reflects a good convergent construct validity. On the contrary, a significant negative correlation was found for the expressive suppression domain between the Ar-CFS and the ERQ (−0.11, *p* = 0.011) and the PSS (−0.45, <0.001). Expressive suppression is a response-focused approach that aims to regulate an emotional experience by inhibiting or suppressing it after it has already been triggered or solidified.

On the other hand, the PSS measures how much a person thinks their life is unpredictable, out of their control, and/or too much to handle. This is different from tools that measure exposure to environmental stress. Both reflect the opposing side of cognitive flexibility, making the negative correlation underscore good discriminant validity. It is important to note that our results support Dennis et al.’s [32] preliminary evidence and Gülüm et al.’s evidence [33] for the convergent construct validity of the cognitive flexibility inventory. This includes correlations with other cognitive flexibility measures and coping strategies.

In this study, we retained all 12 items, including items 3, 4, 6, and 10, to maintain the unidimensional structure of the scale. Although residual correlations between items 4 and 6, and items 3 and 10 were identified, these overlaps reflect natural conceptual connections within cognitive flexibility. Specifically, items 3 and 10 assess decision-making difficulties and knowledge application, while items 4 and 6 capture resilience and creative problem-solving. Retaining these items preserves the comprehensiveness of the tool. The overlaps do not undermine the scale’s validity but instead highlight the interconnected nature of cognitive flexibility. Retaining all 12 items also ensures consistency with the original scale and other validated versions (e.g., Japanese, Turkish, and Spanish) [16,17,18], supporting cross-cultural comparability.

Brown (2015) [34] argued that there could be numerous explanations for a CFA to be misspecified, including omitted item error correlations. Brown added that there should be a rationale for addressing correlational errors in a CFA: “Correlational errors may arise from items that are very similarly worded, reverse-worded, differentially prone to social desirability, or the like”.

A considerable strength of the study is the follow-up duration, which lasted three to six weeks, emphasizing the validity and reliability over time. We included a modest number of patients (110), which, in regular terms, might not be appropriate for CFA. There was also a drastic difference between the two included participant groups. Future studies should consider recruiting more patients and performing CFA for more clinical applicability of Ar-CFS. A weak correlation with ERQ domains and the PSS might raise doubts about the convergent validity. However, they were used due to the unavailability of other Arabic, more cognitive-flexibility-focused measures, which could have captured a better convergent validity. Future studies could further investigate the item relationships (including items 3, 4, 6, and 10) without compromising the tool’s conceptual integrity.

## 5. Conclusions

Our study successfully validated the Ar-CFS to address the significant gap in tools for assessing cognitive flexibility among Arabic speakers. Strong psychometric properties, like high consistency and ICC, showed that the Ar-CFS was reliable for measuring cognitive flexibility in the Saudi population. The findings pointed towards cognitive flexibility being primarily a unidimensional construct. In addition, significant correlations with related psychological constructs further substantiated the scale’s construct validity. To ensure broader applicability, future studies should explore the Ar-CFS across diverse platforms—such as clinical settings, educational institutions, and community contexts—and among varied populations, including physicians, non-medical practitioners, and individuals with different educational backgrounds. This will help confirm the scale’s generalizability across regions and cultural contexts within the Arabic-speaking world.

## Figures and Tables

**Table 1:** Demographic characteristics of participants.

Characteristic	Overall	Student	Patient
	N = 529	N = 419	N = 110
Age	25.8 ± 10.3	21.5 ± 1.6	42.5 ± 12.4
Gender			
Male	228 (43.1%)	197 (47.0%)	31 (28.2%)
Female	301 (56.9%)	222 (53.0%)	79 (71.8%)
Monthly income (SAR)			
<2000 (USD 533)	303 (57.3%)	293 (69.9%)	10 (9.1%)
2000 (USD 533) to <5000 (USD 1332)	85 (16.1%)	70 (16.7%)	15 (13.6%)
5000 (USD 1332) to <10,000 (USD 2665)	49 (9.3%)	12 (2.9%)	37 (33.6%)
10,000 (USD 2665) or more	92 (17.4%)	44 (10.5%)	48 (43.6%)

**Table 2:** Reliability measures and item analysis of the Arabic Cognitive Flexibility Scale.

Internal Consistency						
Item	Mean ± SD	Scale Mean if Item Deleted	Scale Variance if Item Deleted	Corrected Item-Total Correlation	Squared Multiple Correlation	Alpha if Item Deleted
1	4.62 ± 1.042	47.56	44.984	0.492	0.295	0.781
2	3.14 ± 1.274	49.05	45.299	0.351	0.200	0.796
3	4.51 ± 1.313	47.68	42.436	0.513	0.325	0.779
4	4.41 ± 0.928	47.77	46.531	0.438	0.347	0.786
5	3.67 ± 1.331	48.52	47.729	0.187	0.112	0.815
6	4.41 ± 1.008	47.77	43.755	0.613	0.505	0.770
7	4.51 ± 0.958	47.67	44.738	0.568	0.447	0.775
8	4.76 ± 0.902	47.42	46.461	0.461	0.345	0.785
9	4.63 ± 0.862	47.55	46.695	0.467	0.334	0.785
10	3.82 ± 1.287	48.37	43.755	0.442	0.260	0.787
11	4.98 ± 0.875	47.21	47.937	0.350	0.191	0.793
12	4.72 ± 1.070	47.47	43.203	0.611	0.413	0.770
Total 12 items			Scale mean ± SD		Cronbach’s alpha	
			52.19 ± 7.276		0.800	
Test–retest reliability (stability) (N = 36)						
Intraclass correlation		0.82 ** (0.68 to 0.90)			*p* < 0.001	

** Correlation is significant at the 0.01 level (2-tailed).

**Table 3:** Inter-item correlation matrix of the Arabic Cognitive flexibility scale.

	1	2	3	4	5	6	7	8	9	10	11	12
1	1.000	0.182	0.265	0.288	0.064	0.405	0.419	0.267	0.387	0.249	0.277	0.392
2	0.182	1.000	0.297	0.097	0.212	0.259	0.167	0.094	0.129	0.294	0.079	0.321
3	0.265	0.297	1.000	0.271	0.221	0.298	0.317	0.323	0.168	0.417	0.164	0.393
4	0.288	0.097	0.271	1.000	0.024	0.551	0.409	0.317	0.261	0.187	0.158	0.317
5	0.064	0.212	0.221	0.024	1.000	0.089	0.008	0.021	0.043	0.251	0.088	0.083
6	0.405	0.259	0.298	0.551	0.089	1.000	0.515	0.352	0.423	0.233	0.341	0.474
7	0.419	0.167	0.317	0.409	0.008	0.515	1.000	0.495	0.370	0.276	0.241	0.462
8	0.267	0.094	0.323	0.317	0.021	0.352	0.495	1.000	0.405	0.165	0.246	0.365
9	0.387	0.129	0.168	0.261	0.043	0.423	0.370	0.405	1.000	0.180	0.325	0.414
10	0.249	0.294	0.417	0.187	0.251	0.233	0.276	0.165	0.180	1.000	.0113	0.309
11	0.277	0.079	0.164	0.158	0.088	0.341	0.241	0.246	0.325	0.113	1.000	0.309
12	0.392	0.321	0.393	0.317	0.083	0.474	0.462	0.365	0.414	0.309	0.309	1.000

**Table 4:** Confirmatory factor analysis factor loading of the Arabic Cognitive Flexibility Scale.

Component Matrix ^a^	
	Component
	1
#1. I can communicate an idea in many different ways.	0.615
#2. I avoid new and unusual situations.	0.481
#3. I feel like I never get to make decisions.	0.697
#4. I can find workable solutions to seemingly unsolvable problems.	0.531
#5. I seldom have choices when deciding how to behave.	0.243
#6. I am willing to work at creative solutions to problems.	0.766
#7. In any given situation, I am able to act appropriately.	0.657
#8. My behavior is a result of conscious decisions that I make.	0.481
#9. I have many possible ways of behaving in any given situation.	0.516
#10. I have difficulty using my knowledge on a given topic in real-life situations.	0.563
#11. I am willing to listen and consider alternatives for handling a problem.	0.419
#12. I have the self-confidence necessary to try different ways of behaving.	0.757
Extraction method: principal component analysis. ^a^	

^a^. 1 components extracted.

**Table 5:** Validity measures of the Arabic Cognitive Flexibility Scale.

Construct Validity			
Property	Sample		
	Overall (N = 519)	Students (N = 419)	Patients (N = 110)
Emotion regulation (reappraisal)	0.12 ** (0.03 to 0.20) *p* = 0.007	0.12 (0.01 to 0.21) *p* = 0.021	0.14 (−0.05 to 0.32) *p* = 0.164
Emotion regulation (suppression)	−0.11 * (−0.2 to −0.03) *p* = 0.011	−0.11 (−0.19 to 0.01) *p* = 0.025	−0.12 (−0.33 to 0.04) *p* = 0.220
Perceived Stress Scale	−0.45 ** (−0.52 to −0.38) *p* < 0.001	−0.48 (−0.56 to −0.41) *p* < 0.001	−0.25 (−0.43 to −0.07) *p* = 0.010
Content Validity (N = 11)			
Property		Mean ± SD	Min–Max
Scale-level content validity index (S-CVI)		0.925 ± 0.08	0.82–1.0
Simplicity index (SI)		0.895 ± 0.10	0.73–1.0
Importance index (II)		0.910 ± 0.09	0.73–1.0
Clarity index (CI)		0.895 ± 0.11	0.73–1.0

Results are expressed as Pearson’s r coefficient (95% confidence intervals); CI: confidence interval. * Correlation is significant at the 0.05 level (2-tailed). ** Correlation is significant at the 0.01 level (2-tailed).

## Data Availability

Data used in this study are available upon reasonable request from the corresponding author.
